# Biosynthesis and Cytotoxic Properties of Ag, Au, and Bimetallic Nanoparticles Synthesized Using *Lithospermum erythrorhizon* Callus Culture Extract

**DOI:** 10.3390/ijms22179305

**Published:** 2021-08-27

**Authors:** Yury Shkryl, Tatiana Rusapetova, Yulia Yugay, Anna Egorova, Vladimir Silant’ev, Valeria Grigorchuk, Aleksandr Karabtsov, Yana Timofeeva, Elena Vasyutkina, Olesya Kudinova, Vladimir Ivanov, Vadim Kumeiko, Victor Bulgakov

**Affiliations:** 1 Federal Scientific Center of the East Asia Terrestrial Biodiversity, Far Eastern Branch of the Russian Academy of Sciences, 690022 Vladivostok, Russia; avramenko.dvo@gmail.com (T.R.); yuya1992@mail.ru (Y.Y.); kera1313@mail.ru (V.G.); timofeeva@biosoil.ru (Y.T.); levina@biosoil.ru (E.V.); olesya55@list.ru (O.K.); bulgakov@ibss.dvo.ru (V.B.); 2 Department of Molecular Diagnostics and Epidemiology, Central Research Institute of Epidemiology, 111123 Moscow, Russia; bioanna95@list.ru; 3 Department of Biomedical Chemistry, Far Eastern Federal University, 690950 Vladivostok, Russia; vladimir.silantyev@gmail.com; 4 Institute of Chemistry, Far Eastern Branch of the Russian Academy of Sciences, 690022 Vladivostok, Russia; 5 Far Eastern Geological Institute, Far Eastern Branch of the Russian Academy of Sciences, 690022 Vladivostok, Russia; karabzov@fegi.ru (A.K.); dom101@mail.ru (V.I.); 6 Department of Medical Biology and Biotechnology, Far Eastern Federal University, 690950 Vladivostok, Russia; vkumeiko@yandex.ru; 7 A.V. Zhirmunsky National Scientific Center of Marine Biology, Far Eastern Branch of the Russian Academy of Sciences, 690041 Vladivostok, Russia

**Keywords:** green synthesis, silver nanoparticles, gold nanoparticles, Ag/Au nanoparticles, plant cell culture, in vitro, anticancer drugs, biomedical applications

## Abstract

The present study reports a green chemistry approach for the rapid and easy biological synthesis of silver (Ag), gold (Au), and bimetallic Ag/Au nanoparticles using the callus extract of *Lithospermum erythrorhizon* as a reducing and capping agent. The biosynthesized nanoparticles were characterized with ultraviolet-visible (UV-Vis) spectroscopy, X-ray diffraction (XRD) analysis, and transmission electron microscopy (TEM). Our results showed the formation of crystalline metal nanostructures of both spherical and non-spherical shape. Energy dispersive X-ray (EDX) spectroscopy showed the characteristic peaks in the silver and gold regions, confirming the presence of the corresponding elements in the monometallic particles and both elements in the bimetallic particles. Fourier-transform infrared (FTIR) spectroscopy affirmed the role of polysaccharides and polyphenols of the *L. erythrorhizon* extract as the major reducing and capping agents for metal ions. In addition, our results showed that the polysaccharide sample and the fraction containing secondary metabolites isolated from *L. erythrorhizon* were both able to produce large amounts of metallic nanoparticles. The biosynthesized nanoparticles demonstrated cytotoxicity against mouse neuroblastoma and embryonic fibroblast cells, which was considerably higher for Ag nanoparticles and for bimetallic Ag/Au nanoparticles containing a higher molar ratio of silver. However, fibroblast migration was not significantly affected by any of the nanoparticles tested. The obtained results provide a new example of the safe biological production of metallic nanoparticles, but further study is required to uncover the mechanism of their toxicity so that the biomedical potency can be assessed.

## 1. Introduction

Over the past decades, multifunctional metal nanoparticles have become a distinct field of nanotechnology research. The remarkable optical [1,2,3], magnetic [4,5,6], catalytic, and photocatalytic [7,8,9] properties of metal nanoparticles, as well as their antimicrobial, antifungal, and other types of pharmacological activities [10,11,12], have encouraged their use in many areas. Existing and potential applications of metal nanoparticles are spreading across the fields of pharmaceutical therapy [13,14,15], diagnostics [16,17,18], energy conversion [19,20], the food industry [21,22], environmental remediation [23,24], and beyond. The emerging area of nanobiotechnology has resulted in a great demand for methods that can provide rapid, high-throughput, and inexpensive synthesis and which can also be applied at the industrial level. Two major routes have been explored for the synthesis of metallic nanoparticles, namely top-down and bottom-up approaches [25]. The top-down approach assumes the breaking down of bulk material into nanosized structures and usually employs physical methods, whereas the bottom-up approach refers to the *de novo* synthesis of nanoparticles from single atoms, ions, and molecules by chemical or biological catalysts. As an alternative, the green synthesis of metal nanoparticles has recently expanded the bottom-up methods and relies on the utilization of precursors of biological origin that are capable of reducing metal ions into nanoparticles [26]. Compared to conventional technologies, green synthesis avoids ecological and health concerns, has few byproducts, and is cost-effective.

Viruses [27], bacteria and actinomycetes [28,29,30], yeasts and fungi [31,32,33], and algae and plants [34,35,36] have been applied for the synthesis of metallic nanoparticles. Due to the diverse set of bioactive constituents presented in plant extracts, the reduction of metal ions or metal oxides, followed by the nucleation and growth of nanoparticles, occurs in solution at room temperature [37,38,39]. The lack of demand for hazardous chemicals and harsh reaction conditions has made the green synthesis approach an interesting area for further research in the field of nanobiotechnology. To date, nanoparticles made of silver [40,41], gold [42,43], platinum [44,45], palladium [46,47], titanium [48,49], iron [50], zinc, and copper [51,52] have been manufactured. Surprisingly, plants also appear to be capable of nanoparticle formation within the cells [53,54,55,56].

Although over 100 plant species have been studied for their ability to synthesize metal nanoparticles [53,54,55,56], there are only a few reports describing the use of plant cell cultures for this purpose. Plant cell cultures may be explored as simple, low-cost, and eco-friendly manufacturing systems. The first report describing how *Carica papaya* callus can be used for the synthesis of silver nanoparticles (AgNPs) in the range of 60–80 nm was published by Mude and coauthors [57]. Subsequently, metal nanoparticles were obtained using extracts of *Taxus yunnanensis* [58], *Hyptis suaveolens* [59], *Citrullus colocynthis* [60], *Panax ginseng* [61], *Sesuvium portulacastrum* [62], *Linum usitatissimum* [63], *Nicotiana tabacum* [64], and *Solanum incanum* [65] cell cultures. AgNPs and gold nanoparticles (AuNPs) have been produced using *Michelia champaca* calli [66]. Peanut callus promoted the extra- and intracellular reduction of gold ions [67]. Zinc oxide nanoparticles have been produced using extracts of *Viola canescens* callus culture [68]. Thus, the use of plant cell cultures for the fabrication of metal nanoparticles is a new, rapid, and eco-friendly single-step method for the biosynthesis process.

*Lithospermum erythrorhizon* is a herbaceous plant containing a high level of naphthoquinone pigments, mainly shikonin and its derivatives. Due to the remarkable pharmacological activities of naturally occurring compounds, cell cultures of *L. erythrorhizon* have been widely used for the production of oils, gels, and dietary supplements at an industrial scale [69,70]. Its high growth rate, large amount of secondary metabolites (compared to those in the intact roots), and stability of cell lines has encouraged long-term interest in *L. erythrorhizon* in the market place [71]. Here, we report how the cell culture of *L. erythrorhizon* has been used for the production of AgNPs and AuNPs, as well as bimetallic Ag/Au nanoparticles (Ag/Au NPs), using a green synthesis approach. We established parameters affecting the yield and bioactive compounds that can assist the process of bioinspired nanoparticle formation. The cytotoxic activity of metal nanoparticles obtained in the present study was also investigated.

## 2. Results and Discussion

### 2.1. The Influence of Reaction Conditions on AgNPs and AuNPs Biosynthesis

**Figure 1:**
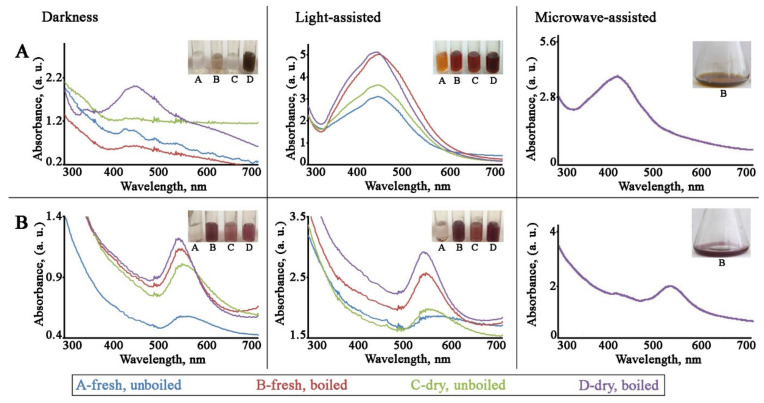
UV-visible spectra of AgNPs (**A**) and AuNPs (**B**) synthesized with *L*. *erythrorhizon* callus extracts under various conditions. The reactions were carried out either in the dark or under continuous illumination. Fresh and dried callus biomass was used for extract preparation. Extracts were boiled or left unboiled before mixing with AgNO_3_ and HAuCl_4_. Biosynthesis was also performed using fresh and boiled extract under microwave irradiation.

We first aimed to estimate the effect of extract preparation (boiled vs. unboiled and fresh vs. dried) and light on the reductive ability of *L*. *erythrorhizon* callus culture. Aqueous extracts of *L*. *erythrorhizon* callus culture, prepared as described in the Materials and Methods section, were mixed with silver nitrate or chloroauric acid solutions, and the reaction mixtures were incubated with or without illumination and with constant rotation. During incubation, the colorless reaction solutions transformed to yellowish-brown and red wine colors for AgNO_3_ and HAuCl_4_, respectively. Both the callus extract without AgNO_3_ or HAuCl_4_ and the metal ion solutions without callus extract showed no change in color. The appearance of the brown and red colors was due to the excitation of surface plasmon vibrations, typical of AgNPs and AuNPs, respectively [41,43], which made it possible to follow the formation of metal nanoparticles in the aqueous solutions by eye. The preliminary investigation of nanoparticle formation was carried out with ultraviolet-visible (UV-Vis) spectroscopic analysis in the range from 300 to 700 nm (Figure 1). The characteristic surface plasmon resonance (SPR) band of reduced AgNPs occurred at 443–445 nm for reactions carried out in all tested conditions. The characteristic SPR band of reduced AuNPs ranged from 538 to 567 nm, depending on the experimental conditions. For example, a blue shift in the SPR peaks was observed for AuNPs prepared with boiled extract from both fresh and dried callus biomass. Similarly, light illumination also provoked a blue shift in the absorption wavelengths of biosynthesized gold nanocrystals. These blue shifts arose due to a decrease in the refractive index of the dielectric environment surrounding the AuNPs and indicated that the average particle sizes were decreasing [72,73]. Interestingly, both the temperature of the extraction procedure and the light conditions of the incubation significantly affected the efficiencies of the reduction reactions (Figure 1). We found that, in general, the boiled extract exhibited a better reductive ability than the unboiled extract in all experimental conditions. Also, reaction mixtures that were incubated in the dark were significantly less intense in color than those that were incubated under illumination. This effect was especially significant in the case of AgNPs, as their formation in the dark was only possible with dried and boiled extract. However, under continuous light illumination, boiling of the extract was more important for the reduction of gold ions. It has previously been shown that the green synthesis of AgNPs in darkness does not produce characteristic SPR peaks [74] even after prolonged reaction times [75]. Moreover, light of different types and spectra, such as UV treatment, sunlight irradiation, and fluorescent light exposure, also promoted the bioinspired synthesis of metal nanoparticles [76,77,78]. Flash light radiation promoted the production of silver, gold, and bimetallic nanoparticles in an energy density-dependent manner [79]. It is possible that some biomolecules, probably aromatic compounds and proteins [80], present in *L*. *erythrorhizon* callus extract become photo-excited under continuous light illumination and release free electrons for the reduction of precursor ions, leading to formation of nanoparticles. It has been shown that photons of blue light have the strongest photocatalytic action compared to other spectral regions [81]. Boiling of the extract is also a commonly used practice for eco-friendly metal nanoparticle biosynthesis [82]. It is generally accepted that this procedure promotes the enrichment of extracts with biological reducing agents, thus enhancing their capacity for the formation of nanocrystals. For example, boiling of the extract was important for conifer-mediated biosynthesis of metallic NPs [83].

Another important factor to consider during the optimization of metal nanoparticles production is microwave irradiation. We tested the potential for microwave heating to intensify silver and gold ion reduction in the presence of dry and boiled *L*. *erythrorhizon* extract. Indeed, microwave treatment for 5 min led to a rapid change of solution, from colorless to yellowish-brown and red wine colors for silver and gold precursors, respectively (Figure 1). However, the productivity of these reactions was compatible with prolonged incubation (for AuNPs), or even lower (for AgNPs). These results indicate that microwave-assisted synthesis has an advantage in terms of the procedure time needed to obtain gold nanostructures, but light irradiation is a more important physical factor than heating for the reduction of precursor ions in the case of AgNP formation. Literature reports have revealed the positive effect of microwave irradiation on the synthesis of metal nanoparticles in both green and chemical approaches. Thus, microwave treatment favored the effective biosynthesis of AgNPs using *Epicoccum nigrum* and *Theobroma cacao* extracts [84,85]. Microwave irradiation also significantly promoted the synthesis of both AgNPs and AuNPs mediated by eosin-methylene blue agar [86]. Moreover, microwave irradiation was sufficient for the preparation of stable AgNPs without the addition of reducing molecules [87].

**Figure 2:**
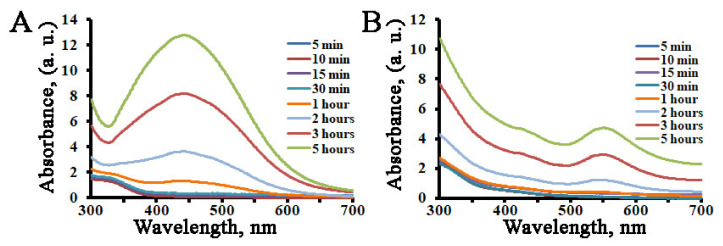
UV-visible spectra of AgNPs (**A**) and AuNPs (**B**) synthesized with *L*. *erythrorhizon* callus extracts as a function of reaction time.

To investigate the reduction of silver and gold ions as a function of time, fresh and boiled extracts of *L*. *erythrorhizon* callus culture were mixed with the corresponding metal ion solution, and the formation of AgNPs and AuNPs was monitored using UV-Vis spectral analysis (Figure 2). In the case of AuNPs, the color of the solution was yellow, and it changed rapidly into a red color within a few minutes of callus extract addition. However, in the case of AgNPs, it took at least 1 h to transform the colorless reaction medium to a pale yellow-brown. It took only 5 h to complete the reduction of both silver and gold ions, and there was no significant change afterwards. No significant shift of the absorption maximums was recorded during the period of incubation. Similar dynamics in color changes were reported previously for AgNPs produced using extracts from *Solanum xanthocarpum*, *Artemisia turcomanica*, *Gleichenia pectinata*, and *Spirogyra varians* [88,89,90,91].

### 2.2. Characterization of the AgNPs and AuNPs

**Figure 3:**
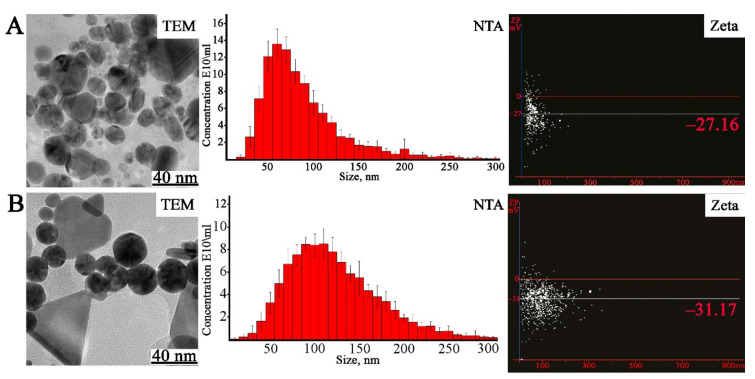
Analysis of AgNPs (**A**) and AuNPs (**B**) synthesized with *L*. *erythrorhizon* callus extract. TEM—transmission electron microscopy images of AgNPs and AuNPs; NTA—nanoparticle tracking analysis; Zeta—measurement of electric potential using NTA.

The shape and size of the biosynthesized AgNPs and AuNPs were studied using transmission electron microscopy (TEM). Analysis of the AgNPs showed the formation of dominantly spherical particles ranging from 20 to 50 nm in size (Figure 3). The difference between the two atomic layers was found to be 0.16 nm. This value corresponds approximately to the interplanar spacing between the (003) planes of silver. TEM images displayed that AuNPs were presented by triangular-, spherical-, and pentagonal-shaped structures in the size range of 10–45 nm (Figure 3). The shortest lattice parameter calculated from each image was 0.23 nm in every case, which corresponds to the interplanar spacing between the (111) planes of gold. The TEM results indicated that the biosynthesized particles were similar to those reported previously [13,77]. In particular, a general trend that AuNPs’ synthesis from plant extracts leads to a variety of shapes and sizes has been previously demonstrated using aqueous solutions derived from jute mallow [92], hibiscus [93], and black tea [94].

The particle size distributions were additionally obtained by nanoparticle tracking analysis (NTA) measurements (Figure 3). The mean size of the synthesized nanoparticles was 85 nm for AgNPs and 108 nm for AuNPs. Notably, the sizes of nanoparticles determined by TEM were much smaller than the values obtained by NTA. It should be taken into account that NTA measurements depend on particle surface properties and indicate the hydrodynamic diameter, whereas TEM can measure the actual diameter of the metal nanocrystals, since the surrounding water molecules are not sufficiently electron dense to provide contrast. Additionally, light scattering techniques, such as NTA, are unable to distinguish individual and agglomerated particles, which may result in a larger calculated diameter than that obtained using TEM. The z-potential of the silver and gold nanostructures was measured using NTA to detect the surface charge of the nanoparticles. The z-potential of AgNPs and AuNPs was found to be −27 mV and −31 mV, respectively (Figure 3). High levels of z-potential cause strong repulsive forces between particles in the colloid solution, which should prevent their agglomeration and thus ensure a high degree of stability [95].

**Figure 4:**
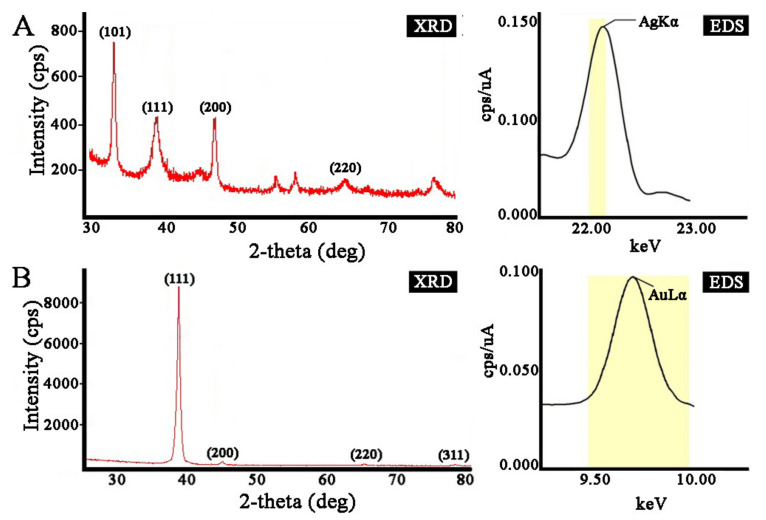
X-ray diffraction (XRD) and energy dispersive X-ray (EDX) analysis of AgNPs (**A**) and AuNPs (**B**) synthesized with *L*. *erythrorhizon* callus extract.

To verify the crystalline nature of the prepared nanoparticles, we used X-ray diffraction (XRD) analysis (Figure 4). The diffraction peaks of AgNPs at the 2*θ* values 38.18°, 44.12°, and 64.55° corresponded to the (111), (200), and (220) lattice planes of a face-centered cubic (fcc) structure, respectively. The corresponding d-spacing values of the AgNPs were 2.36, 2.05, and 1.44, respectively. The diffraction pattern corresponded to almost pure silver metal powder. Similarly, the diffraction peaks at the 2*θ* values 38.24°, 44.48°, 64.71°, and 77.70° corresponded to the (111), (200), (220), and (311) Bragg reflection planes of an fcc structure, respectively, for the Au metallic structure. The d-spacing values of the XRD lines of the AuNPs were 2.35, 2.04, 1.44, and 1.23, respectively. Our data matched with the silver card no. 04-0783 and gold card no. 04-0784 from the database of the Joint Committee on Powder Diffraction Standards (JCPDS). The average grain sizes of the AgNPs and AuNPs formed in our experiments were determined using Scherr’s formula and were estimated to be 8 nm and 14 nm for AgNPs and AuNPs, respectively. The composition of the crystals was further evaluated using energy dispersive X-ray (EDX) analysis, which provided evidence that they consisted of silver and gold without additional impurities (Figure 4). Overall, the obtained results illustrate that the silver and gold ions had indeed been reduced to Ag^0^ and Au^0^ by components of the *L*. *erythrorhizon* callus culture extract under the reaction conditions. The characterization data of these biologically synthesized NPs conformed to those reported earlier by different authors [59,63,67].

**Figure 5:**
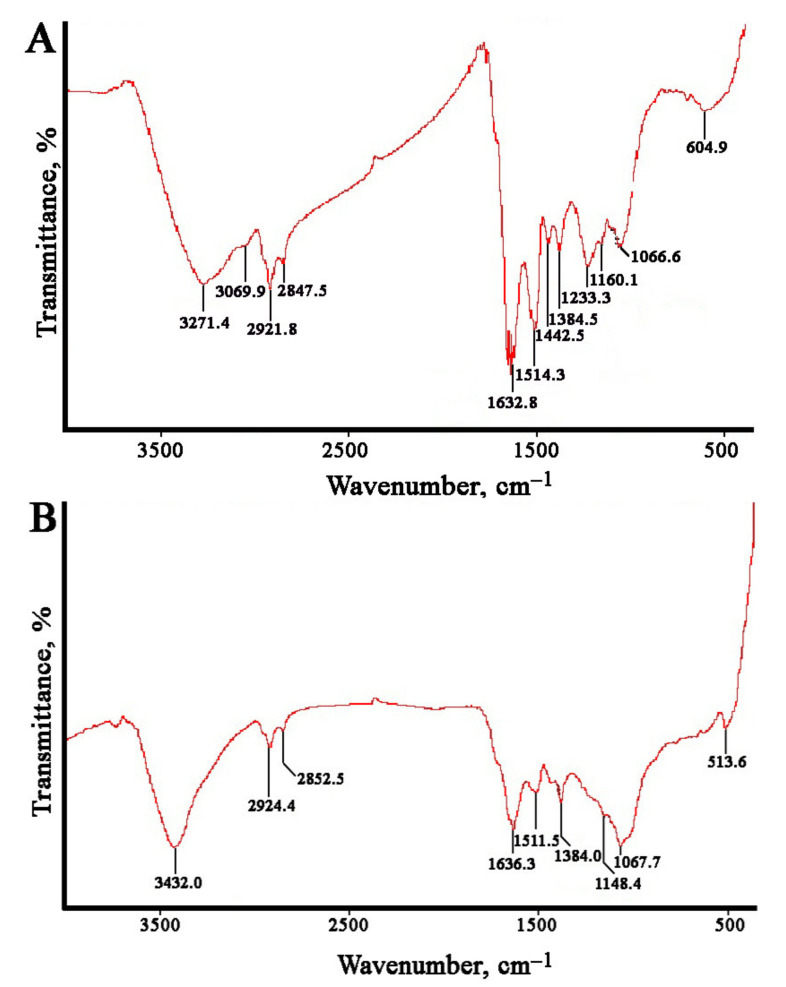
Fourier-transformed infrared (FTIR) spectroscopy analysis of AgNPs (**A**) and AuNPs (**B**) synthesized with *L*. *erythrorhizon* callus extract.

The Fourier-transformed infrared (FTIR) spectroscopy measurements were carried out to estimate the possible biomolecules participating in reduction and stabilization of the metal nanoparticles mediated by the *L*. *erythrorhizon* callus extract (Figure 5). The FTIR spectra of AgNPs showed characteristic peaks at 3271, 3069, 2921, 2847, 1632, 1514, 1442, 1384, 1233, 1160, 1066, and 604 cm^−1^. The AuNPs showed similar peaks at 3432, 2924, 2853, 1636, 1511, 1384, 1148, 1068, and 513 cm^−1^. Large O-H and C-H stretches obtained for silver (3271, 3069, 2921, and 2847 cm^−1^) and gold (3432, 2924, 2853 cm^−1^) nanoparticles are usually attributed to polysaccharides adsorbed on the nanoparticle surface. These bands are also characteristic of the aromatic C-H stretch and O-H stretching of the phenol groups and the carboxylic group [96]. This region also includes N–H stretching vibrations of the amide A and B bands in proteins [97]. In both types of nanoparticles, the main bands were located between 1700 and 1000 cm^−1^. The bands at 1632, 1514, and 1442 cm^−1^ for the AgNPs and those at 1636 and 1511 cm^−1^ for the AuNPs were due to the presence of aromatic rings in the molecule, indicating aromatic ring stretching [98,99]. The peak at 1384 cm^−1^ was attributed to COO− stretching and CH_3_ bending vibrations of lipids and proteins [64]. The absorption bands at 1148–1160 cm^−1^ were mainly attributed to the hydrogen and non-hydrogen bonds of C-O stretching in polysaccharides [100]. In AgNPs, the sample band at approximately 1233 cm^−1^ could be associated with the asymmetric PO_2_^−^ vibration in DNA molecules [101]. The peak at 1067–1068 cm^−1^ was due to the stretching vibrations of C–O–C and C–O bonds in polysaccharides [102]. The remaining bands in the region 600−500 cm^−1^ may have been due to CH out-of-plane bending vibrations of some organic material associated with biosynthesized nanoparticles [102]. Thus, the FTIR spectra of the monometallic nanoparticles showed peaks indicating the presence of polyphenolic compounds, proteins, and polysaccharides from *L. erythrorhizon* callus extract, suggesting that they are involved in the synthesis and stabilization of biogenic nanoparticles. These findings are consistent with data on the biosynthesis of AgNPs and AuNPs using extracts of *piper* [96], industrial hemp [103], and mint [104].

### 2.3. Synthesis of Bimetallic Ag/Au NPs

**Figure 6:**
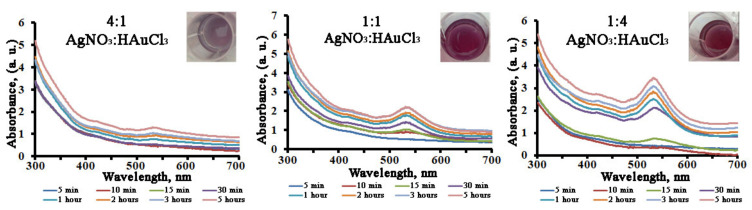
UV-visible spectra of bimetallic Ag/Au NPs synthesized with *L*. *erythrorhizon* callus extract as a function of reaction time.

As *L*. *erythrorhizon* callus extract was capable of promoting monometallic nanoparticle formation, we tested its ability in the production of bimetallic nanocrystals with molar silver/gold ratios of 4:1, 1:1, and 1:4. The biosynthesis of bimetallic Ag/Au NPs after the addition of *L*. *erythrorhizon* callus extract could be followed by a color change from yellow to ruby red (Figure 6) and, further, as a function of reaction time, by UV-Vis spectroscopy for 5 h to complete the reduction of the precursor ions (Figure 6). It was noted that the rate increase of the AuNPs’ SPR band (λ_max_ = 545 nm) was much higher than that of the AgNPs’ SPR band (λ_max_ = 440 nm). Consequently, the AgNPs’ absorbance peak was not visible, even in the Ag/Au (4:1) NPs’ co-reduction mixture. The type of UV-Vis spectra obtained, showing mainly one well-distinguishable SPR peak positioned at the same wavelength as the AuNPs, indicated that the prepared bimetallic NPs may have had a core-shell structure with gold on their shells.

**Figure 7:**
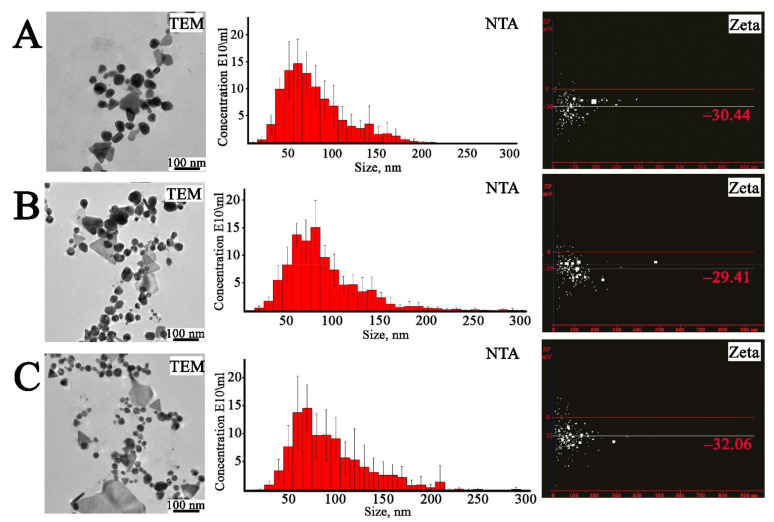
Analysis of bimetallic Ag/Au NPs synthesized with *L*. *erythrorhizon* callus extract. TEM—transmission electron microscopy images; NTA—nanoparticle tracking analysis; Zeta—measurement of electric potential using NTA. (**A**)—Ag/Au (4:1) NPs, (**B**)—Ag/Au (1:1) NPs, (**C**)—Ag/Au (1:4) NPs.

In order to determine the shape and size of the obtained bimetallic nanoparticles, TEM analysis was performed (Figure 7). The Ag/Au NPs in ratios of 4:1 and 1:1 were predominantly spherical and elliptical shapes and, to a lesser extent, triangular, pentagonal, and hexagonal, while the Ag/Au NPs with a ratio of 1:4 were presented mostly as triangular, pentagonal, and hexagonal structures. The sizes of the bimetallic nanoparticles were in the range of 3.0 ± 0.2–45.0 ± 1.3 nm. The difference between the two atomic layers was found to be 0.21 nm. The size distribution of the prepared nanoparticles was also investigated with NTA measurements. The mean sizes of the Ag/Au NPs with 4:1, 1:1, and 1:4 ratios were found to be 89 ± 11 nm, 73 ± 11 nm, and 84 ± 6 nm, respectively. The zeta-potential values for Ag/Au NPs with 4:1, 1:1, and 1:4 ratios were found to be −32.06 mV, −30.44 mV, and −29.41 mV, respectively, indicating the high stability of the bimetallic colloids [105].

**Figure 8:**
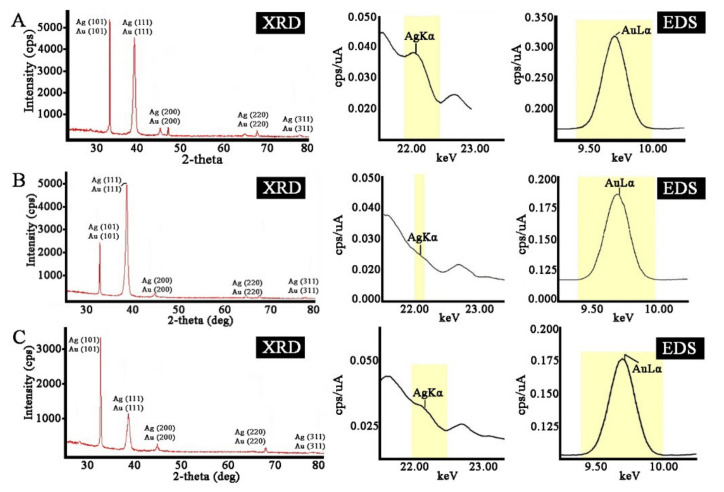
X-ray diffraction (XRD) and energy dispersive X-ray (EDX) analysis of bimetallic Ag/Au NPs synthesized with *L*. *erythrorhizon* callus extract. (**A**)—Ag/Au (4:1) NPs, (**B**)—Ag/Au (1:1) NPs, (**C**)—Ag/Au (1:4) NPs.

The crystalline structure of the biosynthesized bimetallic Ag/Au NPs was evaluated by XRD analysis. As shown in Figure 8, the biogenic nanoparticles showed typical diffraction peaks in the entire spectrum of the 2*θ* values assigned to the (111), (200), (220), and (311) Bragg reflection planes of fcc Ag and Au metallic structures. These XRD results indicate that the prepared bimetallic nanoparticles are fcc structures of silver and gold nanocrystals, similar to those reported previously [96,106]. The calculated crystallite sizes of the nanoparticles were found to be 15, 23, and 18 nm for Ag/Au NPs with 4:1, 1:1, and 1:4 ratios, respectively. The composition of the bimetallic crystals was also studied by EDX analysis, which provided evidence that they consisted of both silver and gold, without additional impurities (Figure 8).

**Figure 9:**
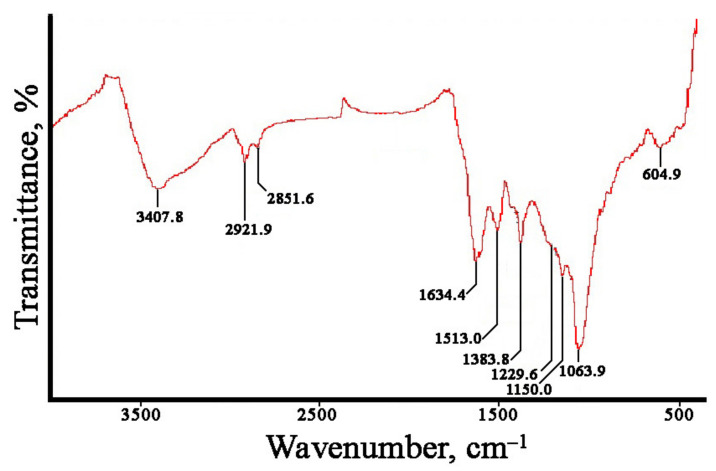
Fourier-transformed infrared (FTIR) spectroscopy analysis of bimetallic Ag/Au (1:1) NPs synthesized with *L*. *erythrorhizon* callus extract.

The FTIR spectra of bimetallic Ag/Au NPs with a 1:1 ratio showed characteristic peaks at 3408, 2922, 2851, 1634, 1513, 1384, 1230, 1064, and 605 cm^−1^. A typical spectrum is shown in Figure 9, and there were no differences between bimetallic nanoparticles with other atomic ratios. It can be noted that the FTIR spectra of Ag/Au NPs resembled those of individual AgNPs and AuNPs synthesized with *L*. *erythrorhizon* callus extract (Figure 5). In particular, the absorption bands at 3407.8 and 1229.6 cm^−1^ were similar to those obtained in AgNPs. Peaks at 1150.0 and 604.9 were characteristic of AuNPs. Other transmittance bands could be attributed to both types of metallic NPs. Thus, our results show that phytochemicals, such as carbohydrates and polyphenolics, as well as proteins, to some extent, play a role in the biological reduction of bimetallic nanoparticles.

### 2.4. The Reducing Activity of L. erythrorhizon Extract Components

The biosynthetic potentials of different plants or cell cultures for the reduction of metal ions differ greatly and evidently depend on the individual biomolecules of extracts that are responsible for metal ion reductions. It seems clear that many different phytochemicals could potentially function as both the reducing and stabilizing agents. Polysaccharides, proteins, and low-molecular weight secondary metabolites are commonly considered to be the major reducing and capping agents present in plant extracts [102,107,108].

**Figure 10:**
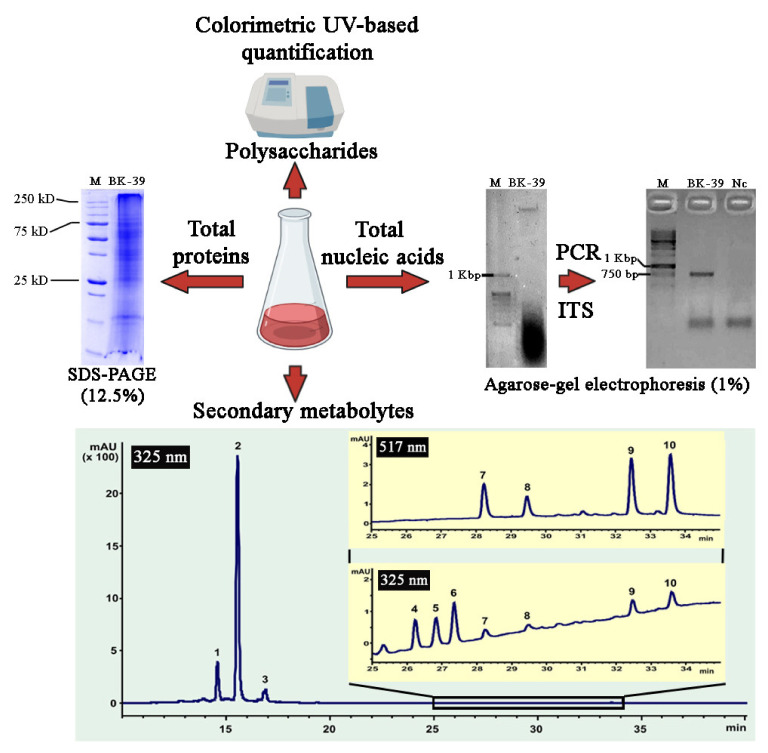
Analysis of nucleic acids, proteins, polysaccharides, and secondary metabolites presented in *L*. *erythrorhizon* callus extract.

**Figure 11:**
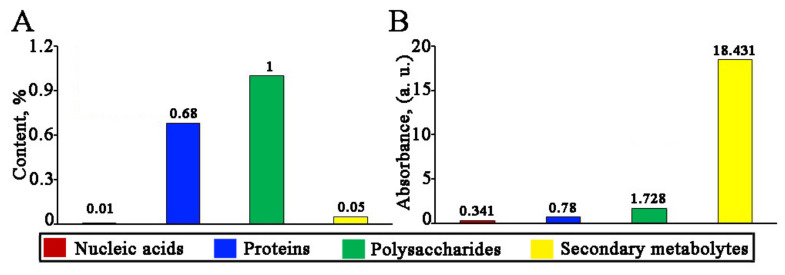
The relative content of nucleic acids, proteins, polysaccharides, and secondary metabolites in *L*. *erythrorhizon* callus extract (**A**) and their reducing activity with respect to silver ions (**B**).

In order to evaluate the roles of the water-soluble molecules, such as nucleic acids, proteins, polysaccharides, and secondary metabolites, of *L*. *erythrorhizon* callus extract in the nanoparticle formation, we analyzed their content and reduction potential in relation to silver ions. Concentrations of nucleic acids, proteins, and polysaccharides were determined using spectrophotometric methods, as described in the Materials and Methods section. DNA/RNA molecules were the least abundant compounds of the extract (Figure 10 and Figure 11). To test the integrity of the DNA sample, a polymerase chain reaction (PCR) was performed with a primer set specific for the plant internal transcribed spacer of nuclear ribosomal DNA. This experiment allowed us to confirm that the *L*. *erythrorhizon* extract contained PCR-compatible DNA as soon as a PCR product of the predicted length was obtained (Figure 10). Proteins presented in the extract of *L*. *erythrorhizon* were analyzed using polyacrylamide gel (PAGE) electrophoresis and the Bradford assay. PAGE electrophoresis revealed that the extract contained a considerable number of proteins that were partially degraded (Figure 10). The Bradford protein assay confirmed the presence of proteins in relatively high concentrations (Figure 11). The polysaccharide fraction extracted from *L*. *erythrorhizon* was the most abundant, as estimated using the phenol-sulfuric acid method (Figure 11). Thus, our results indicated that the main macromolecule presented in the extract was the polysaccharide.

HPLC analysis was further performed to study the fraction of secondary metabolites presented in the extract. The chromatographic profile (Figure 10) for the *L*. *erythrorhizon* extract demonstrated the presence of three major peaks, which were identified as caffeic acid derivatives: rabdosiin (1), rosmarinic acid (2), and methylrosmarinate (3). The remaining minor peaks indicated in Figure 10 were assigned as shikonofuran derivatives—shikonofuran D (4), shikonofuran E (5), and shikonofuran C (6)—and shikonin derivatives—hydroxyisovalerylshikonin (7), acetylshikonin (8), isobutyrylshikonin (9), and isovalerylshikonin (10). All the identified metabolites have been previously detected in *L. erythrorhizon* [109,110,111]. The values of the monoisotopic molecular masses were obtained using high-resolution mass spectrometry, and molecular formulas were assigned to every detected component that displayed a mass error below 0.005 Da. The fragmentation pattern of the precursor ions with composition [M-H]^−^, as well as characteristic absorption maxima obtained from the tops of the indicated peaks, were in good agreement with those published in the literature [109,111,112,113,114] (see Table S1). In general, three main low-molecular weight compounds (rabdosiin, rosmarinic acid, and methylrosmarinate) represented about 0.05% of the total phytochemical content in the *L*. *erythrorhizon* extract.

We further studied the reducing ability of each of the isolated molecular fractions using AgNO_3_ salt. Very low absorption peaks were obtained with the DNA and protein fractions (Figure 11 and Figure S1). At the same time, the polysaccharide sample and the fraction containing secondary metabolites were both able to produce large amounts of AgNPs (Figure 11 and Figure S1). The mean sizes of particles measured with NTA were 111, 84, 89, and 93 nm for nucleic acids, proteins, polysaccharides, and secondary metabolites, respectively. It has previously been shown that proteins may have a reducing and stabilizing effect in the synthesis of silver [115], gold [116], and bimetallic [117,118] nanoparticles. Plant polyphenolic compounds have also demonstrated considerable reducing activity with respect to silver [119] and gold [96] ions or both [120], and polysaccharides have been successfully used to obtain silver [102], gold [121], and bimetallic [121] nanoparticles. As for nucleic acids, these molecules are not usually considered as a reducing agent in green approaches; however, synthetic oligo and polynucleotides are capable of reducing metal ions and are commonly used as flexible templates for metal nanoparticle decoration [122,123]. Overall, our results indicate that phytochemicals extracted from *L*. *erythrorhizon* callus, including high-molecular weight polysaccharides, proteins, and, to a lesser extent, nucleic acids, as well as low-molecular weight polyphenolic compounds, represented by rabdosiin, rosmarinic acid, and methylrosmarinate, exhibit synergistic reduction potential during the biological synthesis of monometallic and bimetallic nanoparticles.

### 2.5. In Vitro Cytotoxicity

**Figure 12:**
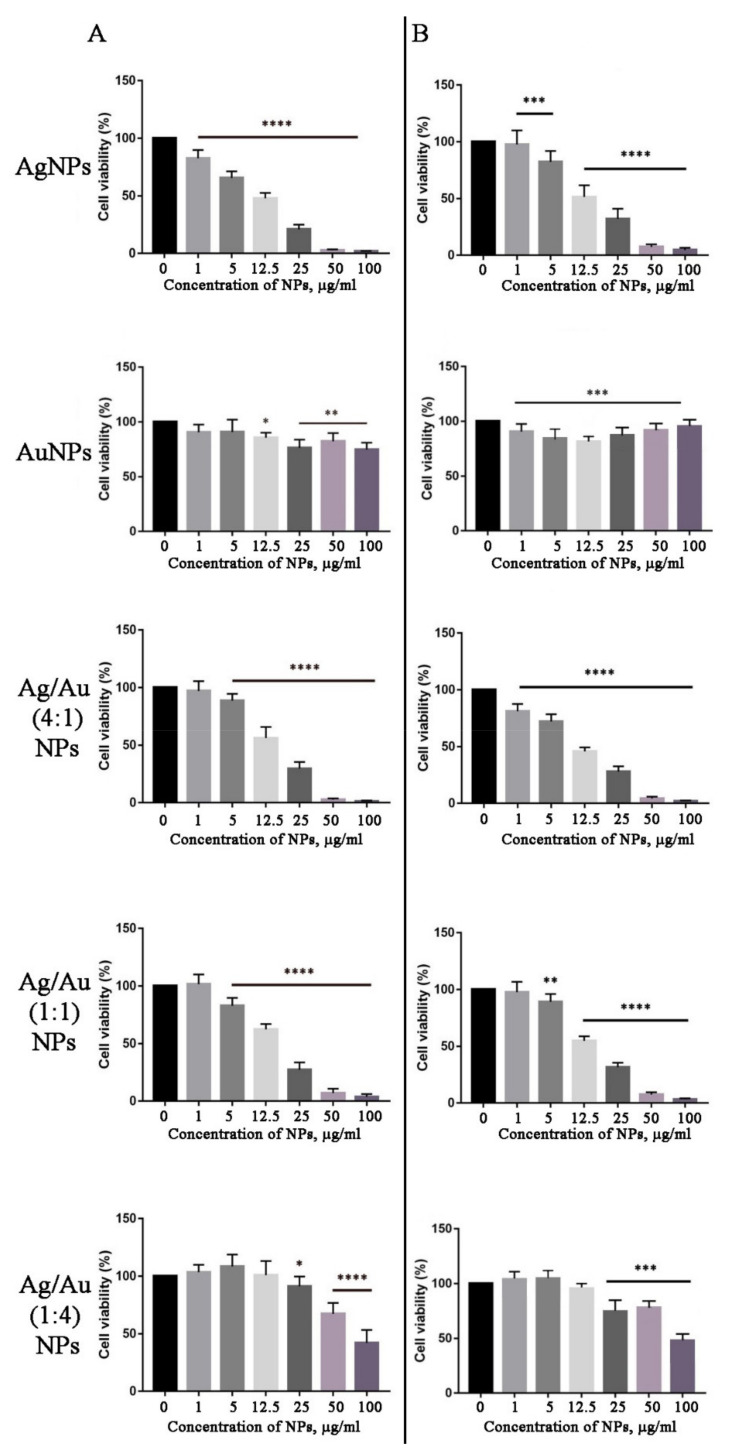
Cell viability of N2A mouse neuroblastoma cells (**A**) and the NIH 3T3 embryonic fibroblast cell line (**B**) in response to AgNPs, AuNPs, and bimetallic Ag/Au NPs synthesized with *L*. *erythrorhizon* callus extract, evaluated by MTT assay. Data are presented as the mean ± SE, * *p* < 0.05, ** *p* < 0.01, *** *p* < 0.001, **** *p* < 0.0001.

The in vitro cytotoxicity of biosynthesized metal nanoparticles was evaluated against mouse neuroblastoma (N2A) and embryonic fibroblast (NIH 3T3) cell lines using the MTT assay. In most cases, the nanoparticles were able to reduce the viability of the N2A and NIH 3T3 cells in a dose-dependent manner, as shown in Figure 12. In particular, treatment of 3T3 and N2A cultures with bimetallic Ag/Au (1:4) and Ag/Au (1:1) NPs did not induce cell death at 1 μg/mL concentration, but significantly (*p* < 0.0001) decreased cell viability at 5 μg/mL and higher doses (Figure 12). Interestingly, under the tested conditions, AgNPs and Ag/Au (4:1) NPs demonstrated the highest cytotoxic effect in both studied cell lines (Figure 12). No significant differences were found in the toxicity of biosynthesized NPs in neuroblastoma and embryonic fibroblast cells. AuNPs possessed the lowest cytotoxicity among the tested NPs (Figure 12). It was previously reported that treatment of MCF7, A549, and Hep2 cell lines with AgNPs, synthesized using *Beta vulgaris* extract, showed a decrease in cell viability with increasing concentrations of AgNPs [124]. The same results were demonstrated when AgNPs obtained with leaf extract of *Andrographis echioides* were studied against MCF7 breast cancer cells [125]. Botha et al. reported that AgNPs and Ag/Au bimetallic nanoparticles, synthesized using *Solidago canadensis* plant extract, showed high toxicity to H4IIE-luc (rat hepatoma) and HuTu-80 (human intestinal) cells, while AuNPs of the same origin were not toxic [118]. Similarly, the *Deinococcus radiodurans* protein extract-mediated AuNPs showed low cytotoxicity against the normal epithelial cells [126]. Interestingly, bimetallic Ag/Au (3:1) NPs synthesized with a chemical reduction had the maximal cytotoxicity effect in cancer cells, compared with Ag/Au (1:1) and (1:3) NPs [127]. Moreover, their toxicity in non-cancerous cell lines was less pronounced and this cell-specific effect was mediated by an excess of tryptophan, which increased the selective toxicity of nanoparticles.

Despite numerous reports describe the cytotoxic activities of metal NPs, the mechanism of their interaction with living cells is complicated and remains not fully understood. It is known that NPs are capable of releasing metal ions in the cytosol and in the nucleus [128]. Such ions readily interact with nitrogen bases and phosphate groups of DNA. Besides, metal ions may interact with the functional groups of proteins within the cell, including those abnormally expressed during tumorigenesis, thereby inhibiting their activity [129]. On the other hand, metal NPs are known to cause oxidative stress, which might represent a primary factor underlying their antitumor activity [130]. AgNPs, AuNPs, and Ag/Au NPs may induce reactive oxygen species production via the Fenton-like reaction [131,132,133,134] followed by protein and lipid oxidation, DNA damage and, consequently, activation of pathways leading to cell death via apoptosis, necrosis, and autophagy [135]. It is generally accepted that AgNPs exhibit higher cytotoxic potency than that of AuNPs. Mukha et al. [136] suggested that this effect is due to a two-fold difference in atomic masses of silver and gold (107.87 and 196.97, respectively). Moreover, cell lines vary in their sensitivity to the same NPs [137].

The concentration, size, shape, surface charge, and surface modifications of metallic NPs also significantly contribute to their mode of action [135]. For example, triangular NPs possess higher biocidal action comparing to spherical NPs [138]. Smaller NPs have been found to cause the strongest dose-dependent toxicity [136,139]. However, very little data are available about the possible correlation between the morphology of bimetallic NPs and their cytotoxic properties. Shmarakov et al. [137] showed that antitumor activity strongly depends on the topological distribution of Ag and Au atoms in alloy and core-shell bimetallic NPs. The most pronounced effects were obtained when a Ag core was covered with a Au shell. Another study demonstrated that the cytotoxic effect of bimetallic NPs was associated with the overall amount of gold [140]. In particular, the cytotoxicity against mouse fibroblasts decreased with the increasing Au mole ratio content in various core-shell compositions [140].

### 2.6. Cell Migration Assay

Based on the cell viability data, AgNPs, bimetallic Ag/Au NPs (4:1), and Ag/Au (1:1) NPs demonstrated the highest inhibitory activity towards NIH 3T3 cell lines, with half-maximal inhibitory concentration (IC50) values of 17, 18, and 17.5 μg/mL, respectively (Figure S2), and these doses were applied for the cell migration assay. It was not possible to estimate IC50 for AuNPs and Ag/Au (1:4) NPs due to their low cytotoxicity, which is why two concentrations, representing an average of the IC50 for other NPs and the highest tested dose (17.5 and 100 μg/mL, respectively), were chosen for the analysis. Both the wound-healing assay and the average speed estimation showed that the tested NPs did not significantly reduce in vitro fibroblast migration in our test conditions (Figure S3, Table S2). It was previously shown that AgNPs obtained using *Manilkara zapota* extract outperformed the cisplatin inhibition of HCT 116 cell migration speed [141]. Surprisingly, the lowest dose of AuNPs induced the most pronounced effect on 3T3 migration speed (Table S2). Similar results were obtained with the human skin fibroblast cell line CCD1072Sk treated with AgNPs and AuNPs [142]. Despite the fact that these NPs did not alter cell viability, both types of nanoparticles were found to reduce cell migration.

## 3. Materials and Methods

### 3.1. Plant Material and Preparation of the Extract

Studies were conducted with a BK-39 callus culture of *Lithospermum erythrorhizon*, which was obtained and described earlier [143]. Calluses were cultivated on solid “W” medium [144], supplemented with 2.0 mg/L kinetin, 0.2 mg/L indole-3-acetic acid, and 0.25 mg/L CuSO_4_, in the dark at 25 °C with 30 day subculture intervals. Freshly collected or dried (60 °C for 5 h) 30 day old BK-39 calli were used to obtain the extracts. Either 1 g of freshly collected or 0.1 g of dried sample was ground in 5 mL of sterile MQ water at room temperature or boiled at 100 °C for 5 min. The resulting broth was centrifuged using a Beckman Coulter Allegra X-22 centrifuge at 20,000× *g*, and the supernatant was finally filtered through a GE Whatman 0.45 µm filter and used for the synthesis of metal nanoparticles.

### 3.2. Synthesis of Monometallic Nanoparticles

Typically, 1 mL of extract was mixed with 9 mL of 1 mM aqueous silver nitrate (AgNO_3_, Sigma-Aldrich, St. Louis, MO, USA) or chloroauric acid (HAuCl_4_, Sigma-Aldrich, St. Louis, MO, USA) solution for the reduction of silver or gold ions, respectively. The reaction was carried out at 25 °C for 24 h and 150 rpm, under continuous light (11 W cool white fluorescent lamp) or in the dark. In the microwave-assisted synthesis, samples were heated for 5 min in a domestic microwave oven (LG MH-6353H) operating at a power of 800 W and a frequency of 2450 MHz. After preparation, the nanoparticles were purified by repeated centrifugation at 20,000× *g* for 20 min, followed by redispersion of the pellet in sterile water.

### 3.3. Synthesis of Bimetallic Nanoparticles

Bimetallic Ag/Au NPs were prepared by mixing a 1:1, 1:4, and 4:1 molar ratios of AgNO_3_ and HAuCl_4_ solutions with 1:10 (*v*/*v*) of boiled BK-39 extract prepared from dried callus biomass. After mixing, simultaneous reduction of both metal ions was carried out at 25 °C for 24 h and 150 rpm, under continuous light (11 W cool white fluorescent lamp). After preparation, the nanoparticles were purified by repeated centrifugation at 20,000× *g* for 20 min, followed by redispersion of the pellet in sterile water.

### 3.4. Characterization of Nanoparticles

#### 3.4.1. UV-Vis Spectrophotometric Analysis

Preliminary evidence of metal nanoparticle formation begins with a visual color change due to SPR effects. Thus, the color change was measured by UV-Vis spectroscopy of sample aliquots (2 µL) drawn from the reaction medium, using a BioSpec-nano spectrophotometer (Shimadzu, Kyoto, Japan) operating at 1 nm resolution with 0.2 mm pathlength.

#### 3.4.2. Fourier-Transform Infrared Spectroscopy Analysis

Fourier-transform infrared (FTIR) spectroscopy of the biosynthesized metal nanoparticles was performed with a FTIR-8400 (Shimadzu, Kyoto, Japan in the range 450–4000 cm^−1^ in KBr pellets at the Laboratory of Micro- and Nanoscale Research at the Far Eastern Geological Institute (FEGI), FEB RAS.

#### 3.4.3. X-ray Diffraction Studies

The XRD patterns for dried samples of nanoparticles were carried out with a Rigaku Miniflex II diffractometer (Rigaku Co., Tokyo, Japan) operating at a voltage of 30 kV and a current of 15 mA, with Cu/Kα radiation in a 2*θ* interval from 3–80°. The average metal nanoparticle size was calculated using the Scherrer equation, as follows:

D = 0.94λ/βCosθ,

where λ is the X-ray wavelength (1.5418 Ǻ), β is the full width at half maximum (FWHM), and θ is the diffraction angle.

#### 3.4.4. Transmission Electron Microscopic Analysis

The samples were characterized by high-resolution TEM. For this analysis, nanoparticles were ultrasonicated in acetone and placed on a carbon-coated copper grid, before being dried at room temperature and used for imaging. TEM was performed using a Libra 200 (Carl Zeiss, Oberkochen, Germany) microscope at the Instrumental Center of Electronic Microscopy at the A.V. Zhirmunsky National Scientific Center of Marine Biology (NSCMB), FEB RAS.

#### 3.4.5. Energy-Dispersive X-ray Spectroscopy Analysis

EDX was used for the elemental analysis of biologically synthesized metal nanocrystals and was performed with a EDX-800HS-P (Shimadzu, Kyoto, Japan) instrument equipped with a Rhodium X-ray tube (settings: vacuum, voltage 50 kV, current 100 µA, measurement time 300 s, dead time 20%, collimator 10 mm). The data were analyzed using PCEDX Shimadzu software.

#### 3.4.6. Nanoparticle Tracking Analysis

The hydrodynamic diameter and zeta potential of nanoparticles were measured by NTA using a NanoSight NS500 system (NanoSight, Amesbury, UK), following the manufacturer’s instructions. Samples were diluted with water to obtain approximately 20 particles per image, before analyzing with the NTA system. The measurements were made at room temperature, with 60 s capture of video clips of particle movement under Brownian motion. The captured videos (10 videos per sample) were then processed and analyzed by NTA analytical software, version 2.2. For zeta-potential estimation, a script for video recording and analysis designed by the manufacturer was employed.

### 3.5. Characterization of the Components of L. erythrorhizon Extract

#### 3.5.1. Isolation and Analysis of Nucleic Acids, Proteins, and Polysaccharides

For DNA precipitation, 3 volumes of absolute ethanol were added to *L*. *erythrorhizon* aqueous callus extract, and the mixture was incubated at –20 °C for an hour. DNA was recovered by centrifugation at 18,000× *g* for 20 min, air-dried, and resuspended in water. DNA samples were electrophoresed through 1% agarose gels containing 0.2 mg/mL of ethidium bromide and visualized under UV light. The DNA concentration was estimated by measuring the absorbance at 260 nm in a microvolume UV-Vis spectrophotometer BioSpec-nano (Shimadzu). To verify the integrity of the DNA, PCR was performed with an undiluted DNA sample and 1:5, 1:10, and 1:50 dilutions of the DNA in water, as previously described [145], using DNA samples obtained from *L*. *erythrorhizon* water extract and an internal transcribed spacer-specific primer pair: ITS-F 5′-GGA AGK ARA AGT CGT AAC AAG G-3′ and ITS-R 5′-RGT TTC TTT TCC TCC GCT TA-3′. Aliquots of PCR reaction products (7 uL) were analyzed using 1% agarose gel electrophoresis.

In order to isolate the bulk fraction protein from *L*. *erythrorhizon* callus extract, aliquots were mixed with 4 volumes of acetone and incubated at –20 °C overnight. Proteins were pelleted by centrifugation (18,000× *g*, 30 min, 4 °C), resuspended in 1X Laemmli buffer, and separated using sodium dodecyl sulfate–polyacrylamide gel electrophoresis (SDS-PAGE) (12.5%) electrophoresis followed by staining with Coomassie brilliant blue G-250. The protein concentration in the samples was determined according to the Bradford protein assay [146]. In brief, 15 day old tissue cultures were frozen and ground in liquid nitrogen, then extracted with 5 volumes of buffer containing 20 mM Tris-HCl, pH 8.0, 8M urea. The homogenates were centrifuged at 14,000× *g* for 20 min, and the supernatant was used to measure the protein concentration. Bovine serum albumin (New England Biolabs, Ipswich, MA, USA) was used for preparation of the calibration curve.

The polysaccharides were precipitated from the *L. erythrorhizon* callus extract overnight using 3 volumes of absolute ethanol. After centrifugation at 18,000× *g* for 20 min, the pellet was resuspended in water and digested for 2 h with 10 U DNaseI at 37 °C, in order to eliminate genomic DNA contamination. The amount of total polysaccharide was measured by the phenol-sulfuric acid method, using a BioSpec-nano spectrophotometer (Shimadzu, Kyoto, Japan) [147].

#### 3.5.2. Chromatography and Mass Spectrometry of Secondary Metabolites

The high-resolution MS data of the identified components of the *L. erythrorhizon* callus culture extracts were recorded using a LCMS-IT-TOF instrument (Shimadzu, Kyoto, Japan) operating in negative ion mode at electrospray ionization conditions. UV-Vis spectra and MS^2^ fragmentation data were obtained based on the Instrumental Centre of Biotechnology and Gene Engineering of IBSS FEB RAS, using a 1260 Infinity analytical HPLC system (Agilent Technologies, Santa Clara, CA, USA) interfaced with a G1315D photodiode array detector and a low-resolution ion trap mass spectrometer (Bruker HCT Ultra PTM Discovery System, Bruker Daltonik GmbH, Bremen, Germany). The chromatographic separation was carried out using reverse phase HPLC columns. A detailed description of the HPLC/UV-Vis/MS methods can be found in the Supplementary Materials. All determined components of the extracts were identified on the basis of UV-Vis and mass spectral data, sequence chromatographic separation using the reverse phase column, and their comparison with the literature data (see the Supplementary Materials). Quantitative determination of the three main components was performed using an external standard of rosmarinic acid, previously isolated by means of liquid chromatography and characterized as described previously [148].

#### 3.5.3. Reduction Ability of *L. erythrorhizon* Extract Components

To evaluate the reduction potential of nucleic acids, proteins, polysaccharides, and secondary metabolites from *L*. *erythrorhizon* extract, the isolated fractions described in Section 3.5.1 were mixed with 1 mM AgNO_3_ in the ratio of 1:9, and the UV-Vis spectrum was recorded after 24 h of incubation to establish the formation of nanoparticles. NTA analysis of the obtained nanoparticles was also performed as described above.

### 3.6. Evaluation of the Cytotoxicity of AgNPs, AuNPs, and Bimetallic Ag/Au NPs

#### 3.6.1. Cell Cultures

Mouse embryonic fibroblast cell line NIH 3T3 and mouse neuroblastoma cell line N2A were cultivated in a mixture of DMEM/F12 growth media (Gibco^®^, Thermo Fisher Scientific, Waltham, MA, USA) combined in a 1:1 (*v*/*v*) ratio, supplemented with 10% fetal bovine serum (FBS), 1× antibiotic-antimycotic solution (Gibco^®^, Thermo Fisher Scientific, Waltham, MA, USA), containing 1000 U/mL penicillin, 100 µg/mL streptomycin, and 0.25 µg/mL amphotericin B, in 75 cm^2^ flasks (Eppendorf, Hamburg, Germany). Cells were incubated at 37 °C in a 5% carbon dioxide humidified incubator upon reaching 70–80% confluence. Cells were washed with PBS (0.8% NaCl, 0.02% KCl, 10 mM Na_2_HPO_4_-KH_2_PO_4_, pH 7.4) and treated with 0.25% trypsin (Gibco^®^, Thermo Fisher Scientific, Waltham, MA, USA). After harvesting, both 3T3 and N2A were seeded in three 96-well plates with each cell culture at 8000 cells per well for cytotoxicity analysis, and 3T3 was seeded in a 24-well plate at 60,000 cells per well for the motility assay. The estimation of cell numbers was performed in a counting chamber.

#### 3.6.2. In Vitro Cytotoxicity Assay

After seeding, 3T3 and N2A cells were left for the next 24 h for adhesion, before varied concentrations of AgNPs, AuNPs, or bimetal Ag/Au NPs (1, 5, 12.5, 25, 50, and 100 μg/mL) were added. The MTT colorimetric assay was performed as described previously [95]. As a control, mouse neuroblastoma and embryonic fibroblasts without nanoparticles were cultured and analyzed. Optical density was measured at 595 nm and 655 nm (background), using an absorbance iMark™ microplate reader (Bio-Rad, Hercules, CA, USA). Each experiment was carried out in eight replicates, and cellular viability was expressed as the percentage of the viable cells to the control.

#### 3.6.3. Cell Migration Assay

The 3T3 cell line was seeded in a 24-well plate in triplicate for each type of experiment until a monolayer was formed and was then treated with varying concentrations of metal nanocrystals. The fibroblast migration assay was performed using high-content imaging based on Cell-IQ with a machine learning platform (CM Technologies Oy, Tampere, Finland). As the drug concentration required to inhibit cellular growth by 50%, the IC50 was calculated from the cell viability data for monometallic AgNPs, as well as for bimetallic Ag/Au (1:1) and Ag/Au (4:1) NPs using GraphPad Prism software version 6.0 (GraphPad Software, La Jolla, CA, USA). The specific concentrations of AuNPs and bimetallic Ag/Au (1:4) NPs used in the assay were 17.5 μg/mL and 100 μg/mL, respectively. Linear wounds were made by scratching with 200 µL-tips (Eppendorf, Hamburg, Germany). Images were taken at a fourfold resolution for 3 h each over 5 days, to allow healing of a ~1500 µm wound at 37 °C and 5% CO_2_. After the wound-healing assay, the average speed of 3T3 treated with different nanoparticles was estimated using a Cell-IQ Analyzer.

### 3.7. Statistical Analysis

All values are expressed as the mean ± standard error (SE). For statistical evaluation, Student’s t-test was used to compare the two independent groups. The level of statistical significance was set at *p* < 0.05. Statistical evaluation was performed using GraphPad Prism software version 6.0 (GraphPad Software, La Jolla, CA, USA).

## 4. Conclusions

A green route for the rapid synthesis of stable AgNPs, AuNPs, and bimetallic Ag/Au NPs using *L. erythrorhizon* callus extract has been demonstrated. The chemical constituents—i.e., polysaccharides, polyphenolic compounds, and proteins—serving as reducing, stabilizing, and capping agents were identified in the extract using various analytical methods. The obtained metal nanoparticles possessed pronounced cytotoxicity but were not able to significantly reduce the number of migrating cells. Without a doubt, further studies need to be conducted to understand mechanisms underlying the toxicity of biosynthesized NPs before their biomedical potential can be estimated. The biosynthesis of nanoparticles using *L. erythrorhizon* callus extract and its components, such as polysaccharides and polyphenols, provides a simple, cost effective, and environmentally friendly route for the synthesis of nanomaterials. The use of well-known biotechnological cell cultures, such as *L. erythrorhizon*, could potentially provide the parallel synthesis of valuable secondary metabolites and nanoparticles within the same biotechnological process.

## Data Availability

All data are contained within the article and Supplementary Materials.

## Supplementary Materials

The following are available online at https://www.mdpi.com/article/10.3390/ijms22179305/s1.
