# Supplementary material for: Biosynthesis and Cytotoxic Properties of Ag, Au, and Bimetallic Nanoparticles Synthesized Using Lithospermum erythrorhizon Callus Culture Extract
Source: Int J Mol Sci. 2021 Aug 27;22(17):9305. doi: 10.3390/ijms22179305 (PMC8431615; doi:10.3390/ijms22179305)
Supplement: Supplementary file 1 [file ijms-22-09305-s001.zip › ijms-1320703-supplementary.pdf]

Supplementary Materials

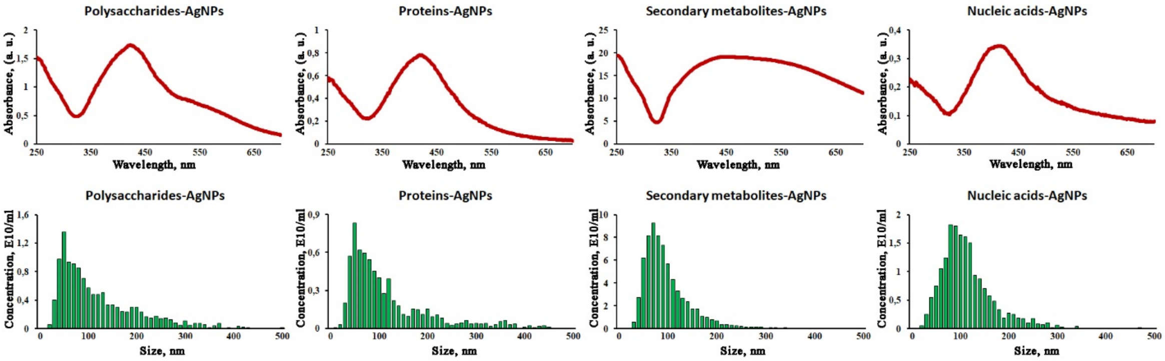

Figure S1. UV-Visible spectra and NTA results of AgNPs synthesized with different fractions of *L. erythrorhizon* callus extracts.

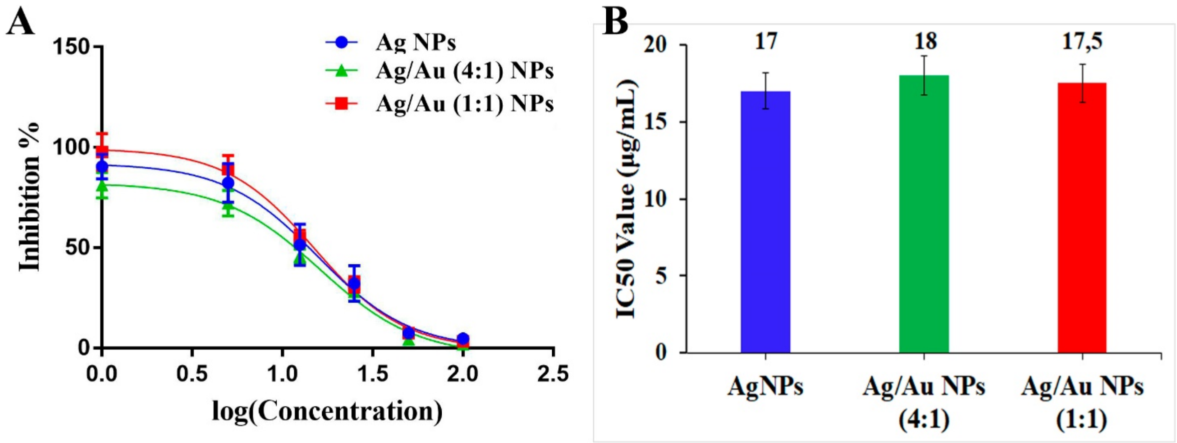

Figure S2. Dose-response curves (A) and half maximal inhibitory concentration (IC50) values (B) of AgNPs, bimetallic Ag/Au NPs (4:1), and Ag/Au NPs (1:1) in mouse embryonic cell line NIH 3T3 based on MTT data. Growth inhibition curves were created by GraphPad Prism software. Cells without NPs treatment were taken as a control. Data are presented as the mean ± SE.

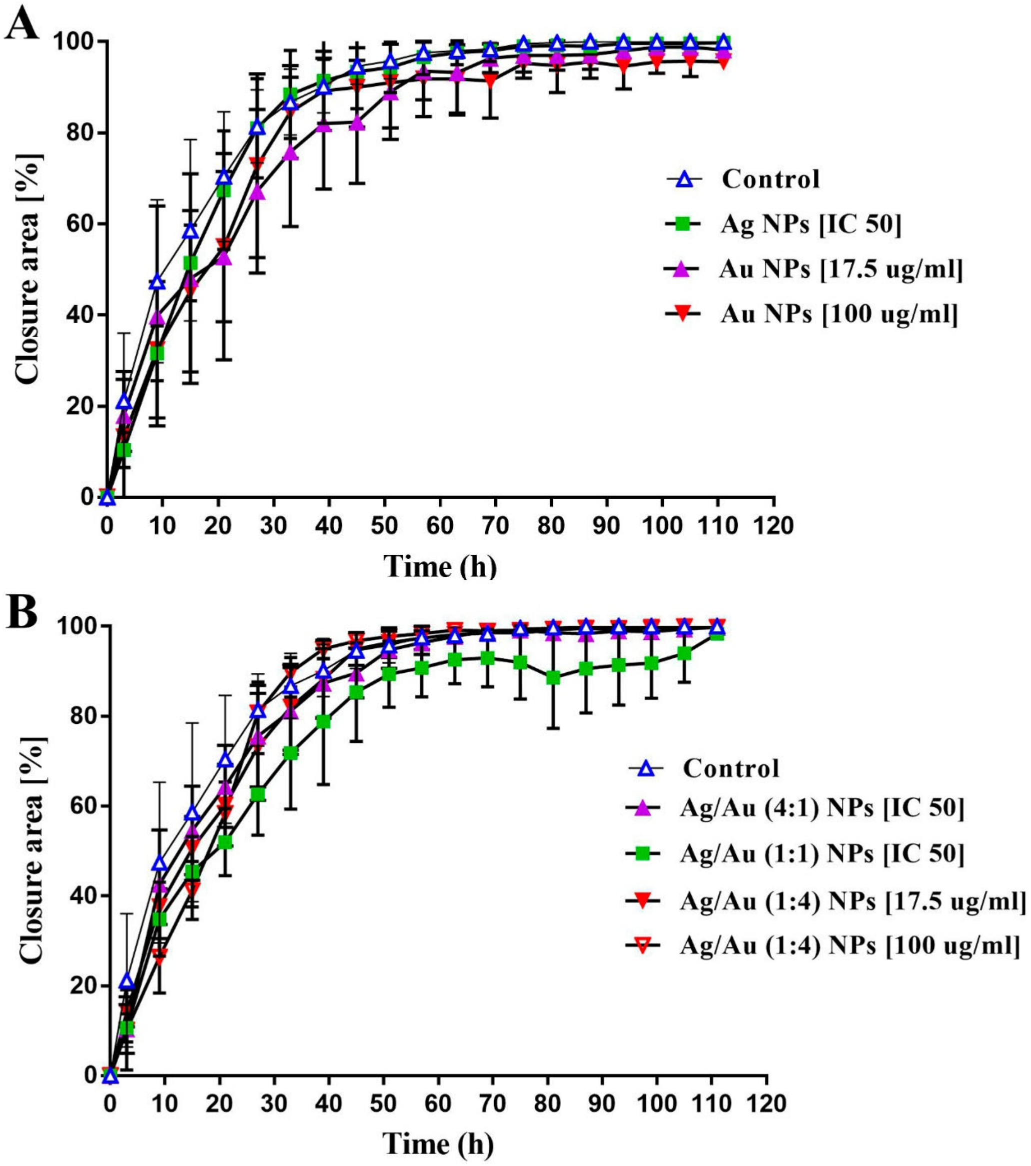

Figure S3. Migration curves of mouse embryonic cell line NIH 3T3 treated with monometallic (A) and bimetallic (B) NPs. 3T3 cells were treated either with IC50 of AgNPs, bimetallic Ag/Au NPs (4:1), and Ag/Au NPs (1:1), or with 17.5 μg/mL and 100 μg/mL of AuNPs and bimetallic Ag/Au NPs (1:4), and were monitored for 5 days. Cells without any treatment were taken as a control. Data are presented as the mean ± SE.

Table S1. Chromatographic and spectrometric data of the main components identified in the extract of *L. erythrorhizon* callus culture.

Peak no.[a]	t_R 1[b] (min)	t_R 2[c] (min)	λ_{max} (nm)	$[M–H]^-$ (*m/z* detected)	$[M–H]^-$ (*m/z* calculated)	Molecular formula	MS^2 fragmentation (precursor ions $[M–H]^-$)[d] (% base peak) (*m/z*)	Putative assignment	Reference
1	9.6	14.6	253, 284, 346	717.1412	717.1461	$C_{36}H_{30}O_{16}$	537(5),519(100),475(29),365(16),339(10)	Rabdosiin	[110, 114]
2	10.5	15.6	287, 328	359.0725	359.0772	$C_{18}H_{16}O_8$	223(6),197(31),179(33),161(100),133(5)	Rosmarinic acid[e]	[110, 112, 113]
3	11.9	16.9	286, 326	373.0880	373.0929	$C_{19}H_{18}O_8$	310(26),285(10),197(28),179(100),175(18),161(38),135(87)	Methylrosmarinate	[113]
4	19.9	26.2	269, 323	343.1517	343.1551	$C_{20}H_{24}O_5$	255(100), 237(5)	Shikonofuran D	[109]
5	20.5	26.8	271, 328	355.1527	355.1551	$C_{21}H_{24}O_5$	273(21), 255(100), 237(3), 227(5)	Shikonofuran E	[109, 110]
6	20.9	27.3	270, 325	357.1662	357.1708	$C_{21}H_{26}O_5$	273(5), 255(100), 237(7), 227(2)	Shikonofuran C	[109]
7	21.5	28.3	272, 518	387.1421	387.1449	$C_{21}H_{24}O_7$	269(100),251(13),117(6)	Hydroxyisovalerylshikonin	[109, 110]
8	22.6	29.5	274, 516	329.1017	329.1031	$C_{18}H_{18}O_6$	269(100),251(30),241(13),225(7)	Acetylshikonin	[109, 110, 111]
9	25.1	32.5	273, 517	357.1318	357.1344	$C_{20}H_{22}O_6$	269(100),251(16),241(3)	Isobutyrylshikonin	[109, 111]
10	26.1	33.6	273, 517	371.1472	371.1500	$C_{21}H_{24}O_6$	269(100),251(38), 241(10),	Isovalerylshikonin	[109, 110, 111]

a The peaks are numbered as shown in Fig. 10

b The retention times of studied components obtained using a LCMS-IT-TOF mass-spectrometer (Shimadzu, Japan)

c The retention times of studied components obtained using an Agilent Technologies 1260 Infinity LC system (Agilent Technologies, USA)

d MS^2 data obtained in low resolution using an ion trap mass spectrometer (Bruker HCT ultra PTM Discovery System, Bruker Daltonik GmbH, Bremen, Germany)

e Reference standard was available

Table S2. Average speed of mouse embryonic fibroblast cell line NIH 3T3 measured with the Cell-iQ® assay*.

Samples	Average speed [µm/h]
NIH 3T3 control (without NPs)	5.06 ± 2.49
NIH 3T3 + AgNPs [IC50]	3.68 ± 1.81
NIH 3T3 + Ag/Au (4:1) NPs [IC50]	3.96 ± 1.49
NIH 3T3 + Ag/Au (1:1) NPs [IC50]	4.19 ± 1.15
NIH 3T3 + Ag/Au (1:4) NPs [17.5 ng/µL]	4.20 ± 1.87
NIH 3T3 + Ag/Au (1:4) NPs [100 ng/µL]	3.21 ± 1.30
NIH 3T3 + AuNPs [17.5 ng/µL]	2.84 ± 0.87
NIH 3T3 + AuNPs [100 ng/µL]	3.27 ± 1.88

*NIH 3T3 cells were treated either with IC50 of AgNPs, Ag/Au (4:1) NPs, and Ag/Au (1:1) NPs or with 17.5 µg/mL and 100 µg/mL of AuNPs and Ag/Au (1:4) NPs. Data are presented as the mean ± SE.

Material and Methods

Analytical chromatography and mass-spectrometry equipment and analysis conditions

The high-performance liquid chromatography with high-resolution mass spectrometry (HPLC-HRMS) analysis of extracts was performed on a Shimadzu Prominence LC (Shimadzu, Kyoto, Japan) equipped with an MS-IT-TOF instrument (Shimadzu, Kyoto, Japan) tandem ion trap/time of flight mass spectrometer. An analytical Ascentis C18 column (100 mm, 2.1-mm i.d., 3-µm part size, Supelco, Bellefonte, Pennsylvania, USA) was used for separation. The column temperature was maintained at 40°C. The binary solvent gradient consisted of 0.1% aqueous acetic acid (A) and acetonitrile (B), with a flow rate of 0.2 mL/min. The gradient started at 0% B, and its percentage was increased to 95% over 25 min. The detection was carried out in negative ion mode and electrospray ionization conditions. The ion source temperature was 200°C, the range of detection was *m/z* 100–1,200, and the potential in the ion source was −3.5 kV. The drying gas (N_2) pressure was 100 kPa. The nebulizer gas (N_2) flow was 1.5 L/min. MS data were collected and processed using the Shimadzu LCMS Solution software (v.3.60.361).

The HPLC/UV-Vis/MS experiments were carried out based on the Instrumental Centre of Biotechnology and Gene Engineering of IBSS FEB RAS using a 1260 Infinity analytical HPLC

system (Agilent Technologies, Santa Clara, California, USA), equipped with a G1315D photodiode array detector. The HPLC system was interfaced with a low-resolution ion trap mass spectrometer (Bruker HCT ultra PTM Discovery System, Bruker Daltonik GmbH, Bremen, Germany) to obtain MS^2 spectra of the detected compounds. The HPLC separation was performed using an analytical Zorbax C18 column (150 mm, 2.1-mm i.d., 3.5-μm part size, Agilent Technologies, USA) at 40°C, with a flow rate of 0.2 mL/min. The gradient started at 0% B, and its percentage was increased to 100% over 30 min. UV-Vis spectra were recorded with a DAD detector in the range 200 - 800 nm. Chromatograms were acquired at wavelengths of 325 nm and 517 nm. The mass spectra were achieved by applying electrospray ionization in negative ion detection. The range of *m/z* detection was 100–1,000, the drying gas (N_2) flow rate was 8.0 L/min, the nebulizer gas (N_2) pressure was 175 kPa, the ion source potential was -4.0 kV, and the drying gas temperature was 325°C. Tandem mass spectra were acquired in Auto-MS^n mode (smart fragmentation) using a ramping of the collision energy. The fragmentation amplitude was set to 1 V. LC data were collected and processed using the Agilent OpenLAB CDS software (v.01.06.111). MS data were collected using the Bruker Daltonics Compass 1.3 esquire control software (v.6.2.581.3) and were processed using the Bruker Daltonics Compass 1.3 Data Analysis software (v.4.0.234.0).
